# Bromopropane Compounds Increase the Stemness of Colorectal Cancer Cells

**DOI:** 10.3390/ijms18091888

**Published:** 2017-09-01

**Authors:** Young-Chang Cho, Thanh Thi Nguyen, So-Yeon Park, Kwonseop Kim, Hyung Sik Kim, Hye Gwang Jeong, Kyung Keun Kim, Hangun Kim

**Affiliations:** 1 College of Pharmacy, Chung-Ang University, 84 Heukseok-ro, Dongjak-gu, Seoul 06974, Korea; cycjjang@hanmail.net; 2 College of Pharmacy, Sunchon National University, 255 Jungang-ro, Sunchon, Jeonnam 57922, Korea; thanhbluesky21@gmail.com (T.T.N.); sinbu17@naver.com (S.-Y.P.); 3 Faculty of Natural Science and Technology, Tay Nguyen University, Buon Ma Thout 630000, Vietnam; 4 College of Pharmacy, Chonnam National University, 77 Yongbong-ro, Buk-gu, Gwangju 61186, Korea; koskim@chonnam.ac.kr; 5 College of Pharmacy, Sungkyunkwan University, 2066 Seobu-ro, Jangan-gu, Suwon 16419, Korea; hkims@skku.edu; 6 College of Pharmacy, Chungnam National University, 99 Daehak-ro, Yuseong-gu, Daejeon 34134, Korea; hgjeong@cnu.ac.kr; 7 Medical Research Center for Gene Regulation, Brain Korea 21 Project, Chonnam National University Medical School, 160 Baekseo-ro, Dong-gu, Gwangju 61469, Korea; kimkk@chonnam.ac.kr

**Keywords:** bromopropane, colorectal cancer, cancer stem cell

## Abstract

Bromopropane (BP) compounds, including 1-bromopropane, 2-bromopropane, and 1,2-dibromopropane, are used in industry for various purposes, and their deleterious effects on human health are becoming known. In this study, we examined the effects of BP compounds on the stemness of colorectal cancer cells. At low, non-cytotoxic concentrations, BP compounds significantly increased spheroid formation in CSC221, DLD1, Caco2, and HT29 cells. In addition, the levels of cancer stem cell markers, such as aldehyde dehydrogenase-1, cluster of differentiation 133 (CD133), CD44, Lgr5, Musashi-1, Ephrin receptor, and Bmi-1 increased after exposure to BP compounds. BP compounds increased the transcriptional activity of the TOPflash and glioma-associated oncogene homolog zinc finger protein (Gli) promoters in reporter assays and increased the expression of Gli-1, Gli-2, Smoothened (SMO), and β-catenin by RT-PCR. These results demonstrate for the first time that BP compounds have the potential to promote cancer stemness.

## 1. Introduction

Colorectal cancer (CRC) is one of the most commonly diagnosed cancers, and is a major cause of cancer deaths in humans worldwide [1,2]. Since the risk factors for CRC, such as unhealthy diet, smoking, and obesity, are becoming more common, CRC incidence rates are rapidly increasing in many countries [3]. Although CRC screening methods and treatments have advanced in recent decades, half of CRC patients experience tumor relapse [4]. The high relapse rate of CRC is thought to be due to the high proportion of cancer stem cells (CSCs), self-renewing cells within tumors [5,6]. CSCs are associated with increased proliferation and invasion and higher rates of tumor relapse and resistance to chemotherapeutics [7,8,9]. Therefore, the discovery of agents that induce cancer cell stemness is critical for the prevention of CRC.

CSCs express specific cell surface markers, including cluster of differentiation 133 (CD133), CD44, aldehyde dehydrogenase-1 (ALDH-1), and leucine-rich repeat-containing G-protein coupled receptor-5 (Lgr-5), and these are also expressed in CRC [10,11,12,13]. Furthermore, several signaling pathways, including the Wnt/β-catenin, Notch, and Hedgehog pathways, which are tightly regulated during embryogenesis and contribute to the pathogenesis of CRC, have been implicated in the development and maintenance of CSCs [13,14,15]. However, the mechanisms that increase the expression of CSC markers and promote CSC generation in CRC are still poorly understood.

Bromopropane (BP) compounds, such as 1-bromopropane (1-BP) and 2-bromopropane (2-BP), have been used as alternatives to ozone-depleting solvents for cleaning metal parts in factories and during the manufacture of fats, waxes, and resins [16]. However, several studies report that these compounds exhibit high toxicity to the reproductive, hematopoietic, central nervous, and immune systems [17,18,19,20]. The Environmental Protection Agency (EPA) has recommended a maximum occupational exposure level of 25 ppm (8 h time-weighted average (TWA)) for 1-BP, to prevent adverse health effects on workers [18]. Furthermore, Korea recommended an occupational exposure level of 1 ppm as a TWA for 2-BP [21]. 1,2-dibromopropane (1,2-diBP), an analog of 2-BP, has been used as an alternative to 2-BP after recognition of the toxic effects of 2-BP. However, 1,2-diBP also induces DNA damage and is still toxic to some organ systems, although its toxic effects are weaker than those of 2-BP [22]. The toxic effects of BP compounds, as described above, were observed at high concentrations; however, the effects of 2-BP at low concentrations (<1 ppm) have not been studied. This study reports the effects of low concentrations of BP compounds on the induction of cancer stemness and the expression of CSC markers in various CRC cell lines. Furthermore, signal transduction pathways involved in the induction of cancer stemness by BP compounds were investigated.

## 2. Results

**Figure 1:**
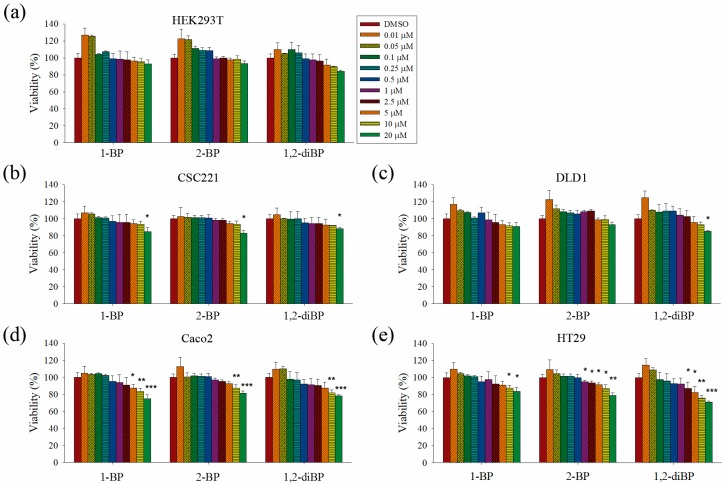
The effects of bromopropane (BP) compounds on the viability of colorectal cancer (CRC) cells. (**a**–**e**): HEK293T (**a**), CSC221 (**b**), DLD1 (**c**), Caco2 (**d**), and HT29 (**e**) cells were treated with various concentrations of 1-BP, 2-BP, or 1,2-diBP for 72 h. After incubation, cell viability was measured as the ability of cells to reduce tetrazolium salts to colored formazan compounds. Relative cell viability compared with the untreated control group for each cancer cell line is shown. Data represent the mean ± standard error of the mean (SEM), and analysis was performed by one-way ANOVA. * *p* < 0.05, ** *p* < 0.01, and *** *p* < 0.001 vs. the untreated control groups for each BP compound.

This study aimed to elucidate whether BP compounds affect the development of cancer stemness. To determine the cytotoxic range of BP compounds on CRC cells, cell viability assays were performed in various CRC cell lines and in HEK293T cells. As shown in Figure 1, BP compounds showed different cytotoxic profiles in various CRC cell lines. The viability of HEK293T cells was not affected by BP compounds. However, BP compounds showed a reduction in cell viability on CRC cell lines, including CSC221, DLD1, Caco2, and HT29 cells. Although each BP compound exhibited a different cytotoxicity profile, none of the BP compounds was cytotoxic at 1 μM (0.123 mg/L for 1-BP and 2-BP; 0.202 mg/L for 1,2-diBP) in any of the CRC cell lines, except for 2-BP, which was cytotoxic to HT29 cells at 1 μM. Since this study aimed to elucidate how BP compounds are involved in cancer stemness at non-cytotoxic low concentrations, and because the above cell viability data showed that BP compounds did not exhibit cytotoxicity concentrations below 1 ppm (1 mg/L), subsequent experiments were mainly conducted with concentrations of BP compounds ≤1 μM. Additionally, a high dose (5 μM) was used in this study to access differences in the regulation of cancer stemness between non-cytotoxic and cytotoxic concentrations of BP compounds.

**Figure 2:**
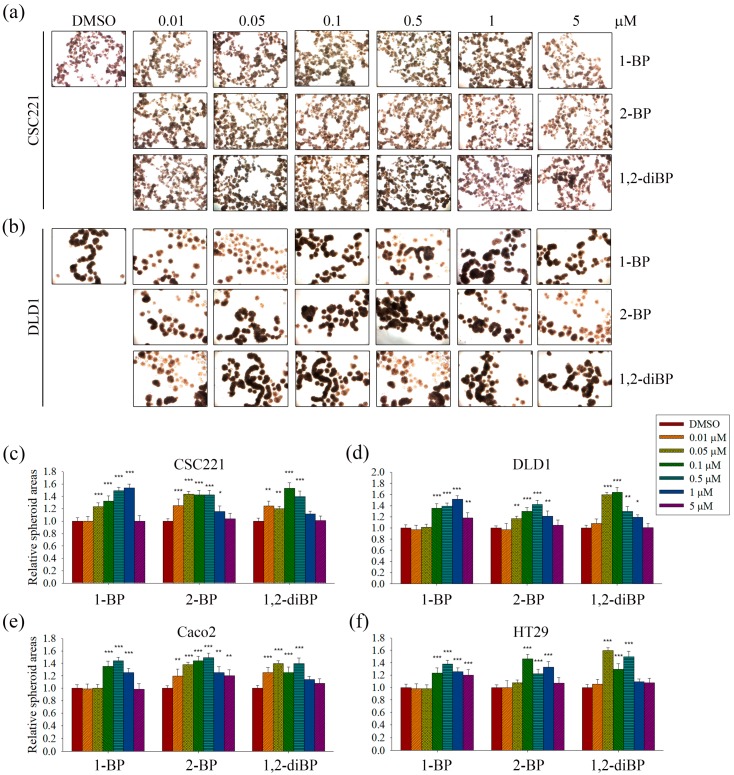
The effects of BP compounds on spheroid formation by CRC cells. CRC cells were treated with various concentrations of 1-BP, 2-BP, or 1,2-diBP for 72 h. After incubation, cells were detached from culture plates and seeded onto 24-well plates coated with a thin layer of 2% low-melting agarose. (**a**,**b**): Spheroid formation in CSC221 (**a**) and DLD1 (**b**) cells at the indicated concentrations; (**c**–**f**) Spheroid formation in CRC cells was calculated as described in Materials and Methods. Relative spheroid formation by each BP compound compared with the untreated control group for each cancer cell line is shown. Data represent the mean ± SEM, and analysis was performed by one-way ANOVA. * *p* < 0.05, ** *p* < 0.01, and *** *p* < 0.001 vs. the untreated control groups for each BP compound.

The induction of cancer stemness by each BP compound was evaluated by measuring spheroid formation in various CRC cell lines. As shown in Figure 2, all BP compounds increased spheroid formation in all CRC cell lines tested, including CSC221, DLD1, Caco2, and HT29 cells; however, their profiles were notably different depending on the dose. 1-BP maximally increased spheroid formation at around 1 μM in all cells. 2-BP increased spheroid formation similarly to 1-BP, but showed maximal effects at around 0.5 μM. In contrast to 1-BP and 2-BP, the effects of 1,2-diBP on spheroid formation were observed at a much lower concentration (0.1 μM). Taken together, these data indicate that BP compounds can increase cancer stemness, although the BP compounds differ with respect to the maximally-effective concentration.

**Figure 3:**
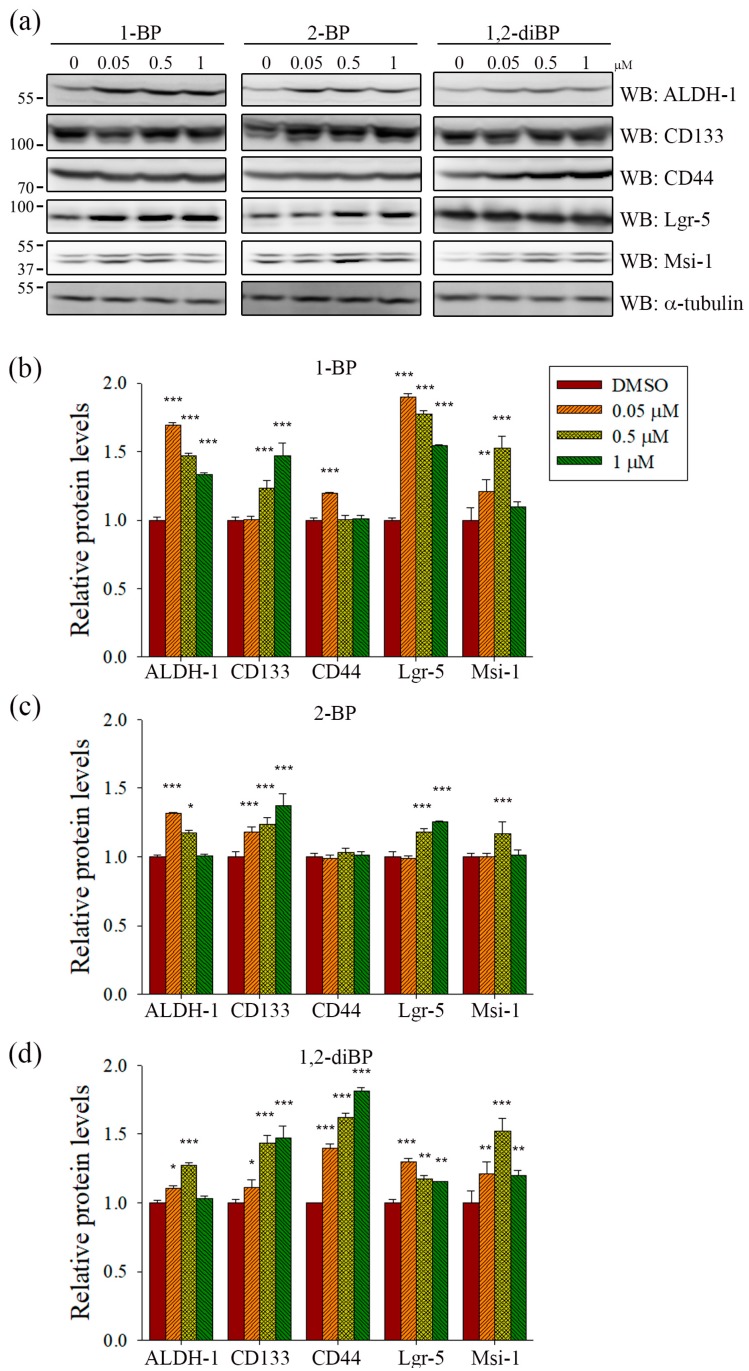
The effects of BP compounds on the expression of proteins related to cancer stemness. CSC221 cells were treated with various concentrations of 1-BP, 2-BP, or 1,2-diBP. After incubation for 72 h, cells were lysed and total protein was subjected to immunoblot analyses with the indicated antibodies. (**a**) Representative immunoblots are shown; and (**b**–**d**) relative levels of each target protein by 1-BP (**b**), 2-BP (**c**), and 1,2-diBP (**d**) compared with the untreated group are shown. Data represent the mean ± SEM, and analysis was performed by one-way ANOVA. * *p* < 0.05, ** *p* < 0.01, and *** *p* < 0.001 vs. the untreated control groups for each target protein.

ALDH-1, CD133, CD44, Lgr-5, and Msi-1 are markers for the acquisition of cancer stemness. Based on the previous experimental data (Figure 2), we hypothesized that each BP compound might increase the expression of CSC markers at the concentrations at which spheroid formation was induced. To investigate whether BP compounds alter the expression of cancer stemness markers in CRC, the protein expression of ALDH-1, CD133, CD44, Lgr-5, and Msi-1 was measured by immunoblot analysis. As shown in Figure 3, each BP compound induced the expression of certain cancer stemness markers.1-BP strongly induced the expression of ALDH-1, CD133, Lgr-5, and Msi-1, but had little effect on CD44. Similarly to 1-BP, 2-BP induced the expression of ALDH-1, CD133, Lgr-5, and Msi-1, but the effects were lower overall than for 1-BP. Changes in the expression of cancer stemness markers were also observed in 1,2-diBP-treated cells, but the effects of 1,2-diBP were distinguishable from those of 1-BP and 2-BP in that 1,2-diBP induced the greatest increase in the expression of CD44 and only moderately induced the other cancer stemness markers. These data suggest that BP compounds could increase cancer stemness by regulating the expression of CSC markers.

**Figure 4:**
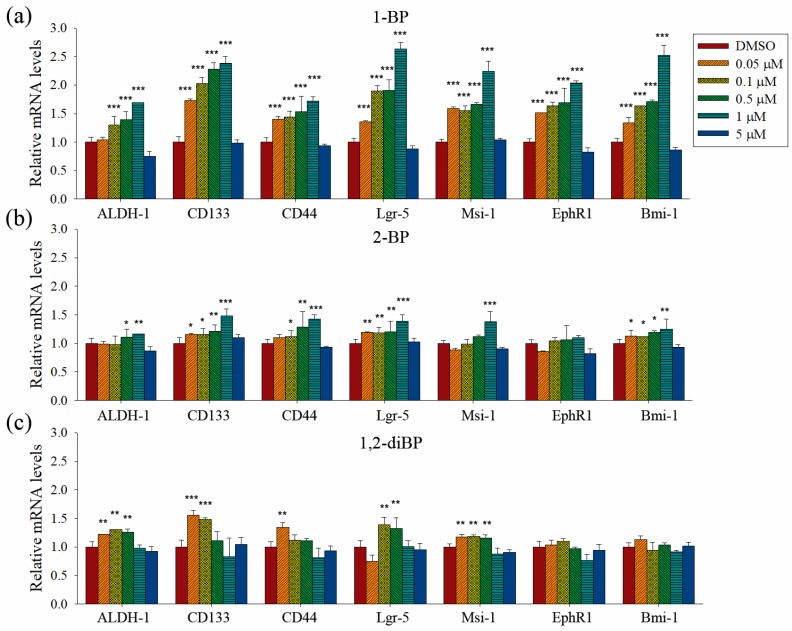
The effects of BP compounds on the mRNA expression of cancer stemness markers. CSC221 cells were treated with various concentrations of 1-BP (**a**), 2-BP (**b**), or 1,2-diBP (**c**) for 48 h. Total RNA was prepared, cDNA was synthesized, and real-time PCR was performed with the primers listed in Materials and Methods. Data represent the relative mRNA expression levels compared with the untreated control groups for each target gene. Data represent the mean ± SEM and were analyzed by one-way ANOVA. * *p* < 0.05, ** *p* < 0.01, and *** *p* < 0.001 vs. the untreated control groups of each target gene.

Quantitative PCR analyses were performed to measure the transcriptional regulation of cancer stemness markers, such as ALDH-1, CD133, CD44, Lgr-5, Msi-1, EphR1, and Bmi-1, by BP compounds (Figure 4). None of the BP compounds induced the expression of cancer stemness markers at 5 μM, indicating that the cancer stemness-inducing effects of BP compounds occur at non-cytotoxic concentrations. All BP compounds increased the mRNA expression of the cancer stemness markers that were upregulated at the protein level in the immunoblot analyses, although minor differences were observed in the transcriptional regulation of each marker. 1-BP increased all cancer stemness markers in a dose-dependent manner. In contrast to the immunoblot data, 1-BP and 2-BP increased the expression of CD44 mRNA. However, the relative changes in CD44 mRNA levels induced by 1-BP and 2-BP were less than the relative increase in CD44 protein levels induced by 1,2-diBP. Compared with 1-BP, 2-BP, and 1,2-diBP only moderately increased the mRNA expression of cancer stemness markers. Overall, the effects of BP compounds on the mRNA expression profiles of CSC markers were similar to the effects observed in the spheroid formation assay. These data demonstrate that BP compounds enhance the transcription and protein expression of CSC markers in CRC cell lines, indicating that these compounds could increase the CSC population in these cells.

**Figure 5:**
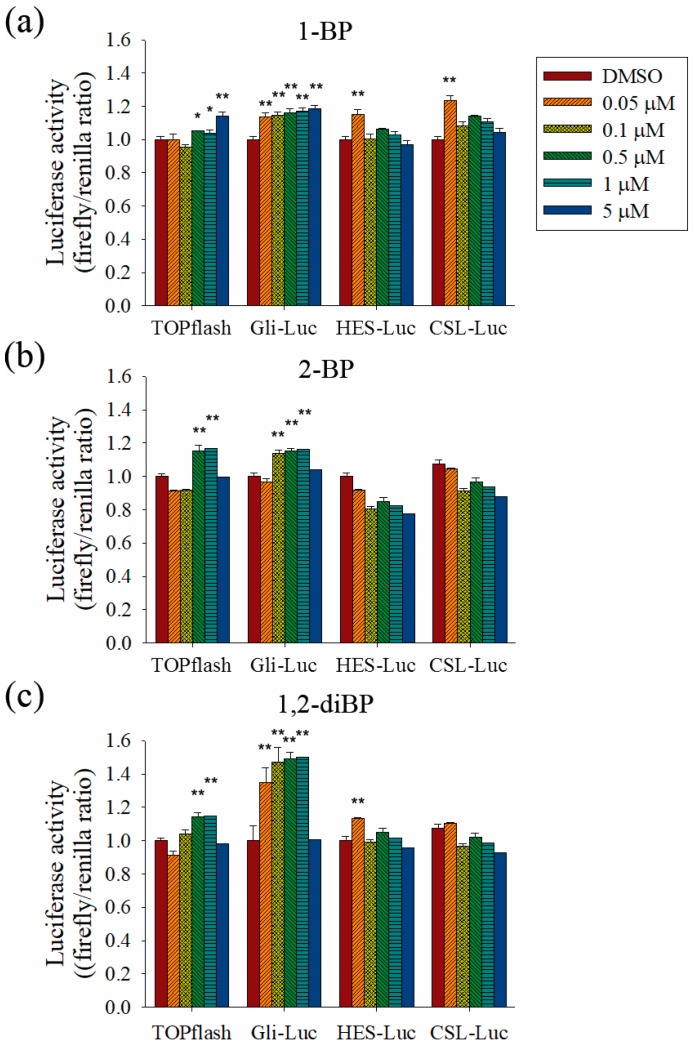
The effects of BP compounds on the activities of promoters related to Hedgehog, Notch, and Wnt signaling. HEK293T cells were co-transfected with the pRL-TK (renilla) plasmid and the pTOPflash, pGli-luc, pHES-luc, or pCSL-luc reporter plasmid (firefly). After 24 h, transfected cells were treated with various concentrations of 1-BP (**a**), 2-BP (**b**), or 1,2-diBP (**c**) and incubated for an additional 48 h. Cells were lysed, and firefly and renilla luciferase activity was measured. The firefly luciferase activity of each group was calculated, using the renilla luciferase activity as an internal control. The relative firefly luciferase activity of each BP compound compared with the untreated control group is shown. Data represent the mean ± SEM, and were analyzed by one-way ANOVA. * *p* < 0.05, ** *p* < 0.01, and *** *p*< 0.001 vs. the untreated control groups for each BP compound.

To evaluate the role of the Hedgehog, Notch, and Wnt signaling pathways on the increased transcription of CSC markers observed in response to BP compounds, the effect of BP compounds on the activity of promoters such as TOPflash, Gli, hairy/enhancer of Split (HES), and CBF1/Su(H)/Lag-1 (CSL) was measured (Figure 5). BP compounds mainly enhanced the activation of promoters, such as TOPflash and Gli, which are activated by Wnt and Hedgehog signaling-related transcriptional factors. However, activation of the HES and CSL promoters was induced by 1-BP and 1,2-diBP at a concentration of 0.05 μM. These data support the hypothesis that the induction of cancer stemness by BP compounds occurs via the Hedgehog, Notch, and Wnt signaling pathways.

**Figure 6:**
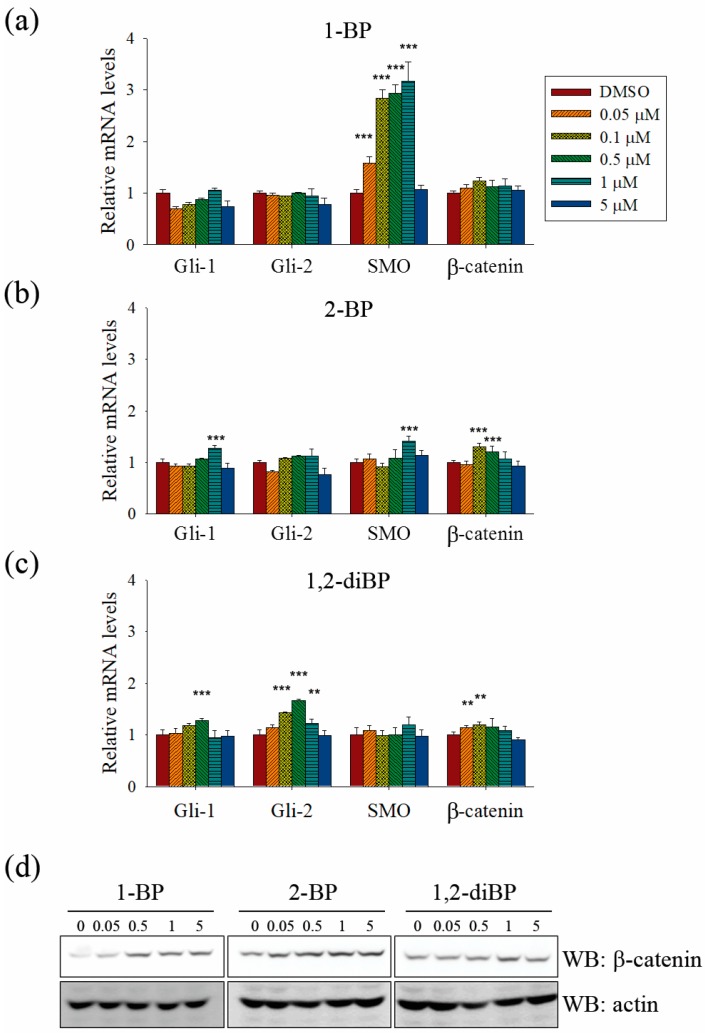
The effects of BP compounds on the expression of transcription factors related to the Hedgehog, Notch, and Wnt signaling pathways. CSC221 cells were treated with various concentrations of 1-BP, 2-BP, or 1,2-diBP for 48 h (**a**–**c**) or 72 h (**d**). (**a**–**c**): Total RNA from 1-BP- (**a**), 2-BP- (**b**), or 1,2-diBP-treated cells (**c**) were prepared, cDNA was synthesized, and real-time PCR was performed with the primers listed in Materials and Methods. Data represent the relative mRNA expression levels compared with the untreated control groups for each target gene. Data are shown as the mean ± SEM and were analyzed by one-way ANOVA. * *p* < 0.05, ** *p* < 0.01, and *** *p* < 0.001 vs. the untreated control groups for each target gene. (**d**) Cell lysates were subjected to immunoblot analyses with β-catenin antibody.

Further experiments were performed to confirm whether BP compounds induce the activation of Hedgehog and Wnt signaling pathways. The expression of signaling molecules, such as Gli1, Gli2, SMO, and β-catenin, was measured by RT-PCR in BP compound-treated CSC221 cells. As shown in Figure 6a–c, a moderate increase in the expression of β-catenin mRNA was observed in BP compound-treated CSC221 cells. Moreover, it was observed that the protein levels of β-catenin were increased by BP compounds treatment in CSC221 cells (Figure 6d), and, together, these might explain the increase in TOPflash promoter activity induced by BP compounds. Furthermore, the notable increase in Gli promoter activity induced by BP compounds might be due to the significant increase in SMO induced by 1-BP and the increase in Gli-1 and -2 induced by 2-BP and 1,2-diBP (Figure 6a–c). The notable increase in HES promoter activity induced by 1-BP and 1,2-diBP at 0.05 μM correlated with the increased mRNA expression of SMO and Gli-2 in response to 1-BP and 1,2-diBP, respectively, at that concentration (Figure 6a–c). Collectively, these data indicate that transcription factors involved in the Hedgehog and Wnt signaling pathways are regulated by BP compounds.

## 3. Discussion

The scientific literature on the toxicity of BP compounds was previously limited to studies of its cytotoxic effects, and little information was available on the additional toxic effects of these compounds. Here, we evaluated the cancer stemness-inducing effects of various BP compounds, including 1-BP, 2-BP, and 1,2-diBP, on CRC cell lines using sub-cytotoxic concentrations. The major findings were as follows: (1) BP compounds increased spheroid formation at low concentrations in CRC cells; (2) BP compounds increased the expression of cancer stemness markers at both the mRNA and protein levels; and (3) BP compounds increased the transcriptional activity of the Hedgehog, Notch, and Wnt signaling pathways. These data demonstrate that BP compounds induce cancer stemness at low concentrations and could inform new guidelines on the safe use of these compounds.

The induction of spheroid formation in CRC cell lines by 1-BP occurred relatively late compared with the increase in spheroid formation induced by 2-BP and 1,2-diBP (Figure 2). This finding is somewhat difficult to understand, since 1-BP increased the activity of all cancer-related promoters tested. Furthermore, the expression of cancer stemness markers and signaling molecules that are regulated by the transcriptional activity of Hedgehog, Notch, and Wnt was also increased by 1-BP. This unexpected result may be due to the activation of CSL promoter activity by 1-BP at low concentrations. Previous reports indicate that CSL decreases, rather than increases, Notch signaling [23,24,25]. Therefore, it is possible that the activation of CSL by 1-BP at low concentrations could oppose the activation of the other cancer stemness-inducing signals, at least in part.

The relationship between BP compounds exposure and human cancer has not been reported from human studies. Increased lung, large intestine and skin cancers were observed in rodents inhalationally exposed to 1-BP, but the exact mechanism how 1-BP causes cancer remains elusive [26]. In this study, we showed that BP compounds increase the expression of cancer stemness markers in the CSC221 CRC cell line (Figure 3 and Figure 4). Furthermore, we suggested that the induction of cancer stemness by BP compounds might occur via the activation of Hedgehog, Notch, and Wnt signaling molecules (Figure 5 and Figure 6). Many compounds have been reported to activate the above signaling pathway, and these could increase cancer stemness. For example, Sims-Mourtada et al. reported that docetaxel-induced nuclear accumulation of Gli-1 leads to activation of Hedgehog signal and expansion of breast cancer stem-like populations [27]. Chang et al. reported that canonical and noncanonical Wnt signalings are activated by chitosan, and colon and hepatocellular carcinoma cells cultured on chitosan showed increased cell motility, drug resistance, quiescent population, self-renewal capacity, and the expression levels of stemness and CSC marker genes [28]. Likely, Martins-Neves et al. reported that conventional chemotherapy induces stemness in osteosarcoma cells through activation of Wnt/β-catenin signaling [29]. Interestingly, it was reported that 1-BP inhibits glycogen synthase kinase-3β (GSK-3β) by phosphorylating Ser-9 residue on GSK-3β [30]. Given the fact that ubiquitination and proteosomal degradation of β-catenin is mediated by GSK-3α/β, the accumulation of β-catenin and the activation of TOPflash by BP compounds in our results could be partly explained. However, of note is that it is difficult to completely attribute the increased cancer stemness to the activation of these signaling molecules, since other signaling pathways, including the TGF-β and Hippo pathways, are also involved in the regulation of cancer stemness [31,32,33,34]. Further studies using inhibitors that target cancer stemness marker-inducing signaling pathways could clarify the main signaling pathways involved in the induction of cancer stemness markers in response to BP compounds.

Our study identified the carcinogenic effects of BP compounds at low concentrations. The effects of BP compounds on cancer stemness can be through affecting related signaling pathways directly or, alternatively, can be because cancer stem cells are resistant to BP compounds. Thus far, one of the two hypotheses cannot be ruled out by our data but, rather, the two hypotheses can double the effects of each other and cause a synergistic effect. In fact, it is well known that CSCs are enriched in drug-transporter proteins, thus, protecting themselves from further mutations [35]. Therefore, if BP compounds itself possess stemness-promoting property, exposure to BP compounds will increase the stemness of the cancer cells and subsequently enhances the resistance to the cytotoxic substances through the enrichment of drug-transporters. For the latter possibility, if cancer stem cells are resistant to BP compounds than differentiated cancer cells, exposure to BP compounds, in turn, will increase cancer stem cell populations. Collectively, in either case, favorable microenvironments that produce cancer are created by BP compounds. Collectively, this study aimed to characterize the carcinogenic effects of BP compounds at low concentrations. Here, we show that BP compounds increase cancer stemness by inducing cancer-related signaling pathways, including Hedgehog, Notch, and Wnt. Comparisons of the effects of high and low doses of BP compounds on cancer development in animal models might be required to confirm the different toxic effects of BPs at different doses. From present studies, it appears that increases in stemness are considered as a potential initial step for intestinal carcinogenesis. However, as carcinogenesis is a multistep process and a protracted sequence of additional events are required to produce a fully-malignant tumor, in which stages of initiation and/or progression during cancer development are affected by BP compounds should be demonstrated in experimental models of carcinogenesis. Regardless, we believe that our findings provide the scientific community with key information that helps characterize cancer stemness-inducing effects of BP compounds.

## 4. Materials and Methods

### 4.1. Cell Culture and Reagents

HEK293T (human embryonic kidney), Caco2, DLD1, and HT29 (colorectal cancer) cell lines were purchased from the American Type Culture Collection (ATCC, Manassas, VA, USA). CSC221 (human colorectal adenocarcinoma-enriched cancer stem cell) cell line was purchased from BioMedicure (San Diego, CA, USA) [36]. Known genetic characteristics of CRC cell lines used in this study are as follows [37,38]: Caco2 has a mutation on TP53 and APC; DLD1 has a mutation on KRAS, BRAF, PIK3CA, TP53, and APC; HT29 has a mutation on BRAF, PIK3CA, TP53, and APC. HEK293T, Caco2, DLD1, HT29, and CSC221 cells were maintained in DMEM supplemented with 10% fetal bovine serum (FBS) and 1% penicillin/streptomycin at 37 °C in a 5% CO_2_ atmosphere. BP compounds were purchased from Sigma Chemical Co. (St. Louis, MO, USA).

### 4.2. MTT Assay

Cells were seeded in a 96-well plate were treated with 1-BP, 2-BP, and 1,2-diBP for 60 h. When the former came to the completion of the treatment duration, cultures were supplemented with 2*H*-tetrazolium, 2-(4,5-dimethyl-2-thiazolyl)-3,5-diphenyl-, bromide (MTT). After the incubation at 37 °C, the cells were lysed with lysis buffer containing 50% of dimethylformamide and 20% sodium dodecyl sulfate (SDS) and absorbance was measured at 570 nm using a microplate reader (VERSAmax, Molecular Devices, Sunnyvale, CA, USA). The percentage of cell viability was calculated using the following formula:

Percentage cell viability = (OD of the experiment samples/OD of the control) × 100%

IC_50_ was calculated by SPSS software version 17 (International Business Machines Corp., Armonk, NY, USA).

### 4.3. Spheroid Formation Assay

At ~70% confluence, monolayer cells were dissociated with trypsin-ethylenediaminetetraactic acid (EDTA) into single-cell suspensions. The cells were then inoculated into N_2_ supplemented DMEM/F12 (Invitrogen, Carlsbad, CA, USA) containing human recombinant epidermal growth factor (hrEGF; 20 ng/mL; Biovision, Milpitas, CA, USA) and human basis fibroblast growth factor (hbFGF; 10 ng/mL; Invitrogen). 1-BP, 2-BP, 1,2-diBP, or DMSO (0.01%) as a control were added to the cells at a density of 1 × 10^4^ cells/well in ultra-low attachment 24-well plates (Corning Inc., Corning, NY, USA). After 10–14 days culture, spheres were quantitated by inverted phase contrast microscopy (Nikon Instech Co., Ltd., Kawasaki, Japan). Pixel intensity of sphere area was measured by IMT iSolution software version 21.1 (IMT i-Solution Inc., Northampton, NJ, USA) from random microscope views in each plate. To measure the percent area of a sphere, the pixel amount of the sphere area was normalized by a given pixel × pixel square. Data represent the average of three experiments.

### 4.4. Immunoblot Analysis

Cells were treated with various concentrations of BP compounds for 72 h to detect cancer stem cell markers, as previously described [39]. Mouse anti-ALDH-1 (Santa Cruz Biotechnology, INC., Dallas, TX, USA), rabbit anti-CD133 (Cell applications, INC., San Diego, CA, USA), mouse anti-CD44, and rabbit anti-β-catenin (Cell Signaling Technology, Danvers, MA, USA), rabbit anti-Lgr-5, and rabbit anti-Msi-1 (Abcam, Cambridge, England) were used as primary antibodies for the detection of cancer stemness markers. Rabbit anti-α-tubulin (Cell signaling Technology) was used as an internal standard. All results are representative from at least three independent experiments. Bands were measured by Multi-Gauge 3.0 (Fuji photo film Co., Ltd., Tokyo, Japan) and their relative density calculated based on the density of the α-tubulin bands in each sample. Values were expressed as arbitrary densitometric units corresponding to signal intensity.

### 4.5. qRT-PCR

One microgram of total RNA from each group of treated cells was converted to cDNA with a M-MLV reverse Transcriptase kit (Invitrogen) and SYBR green (Enzynomics, Daejeon, Korea). The primers used for the real-time PCR were ALDH1 (forward) 5′-tgt tag ctc atg ccg act tg-3′ and (reverse) 5′-ttc tta gcc cgc tca ac act-3′ (product size, 154 bp); EphB1 (forward) 5′-tgc aag gag acc ttc aac ct-3′ and (reverse) 5′-cgg tgt tga ttt tca tga cg-3′ (147 bp); Msi-1 (forward) 5′-acc aag aga tcc agg ggt tt-3′ and (reverse) 5′-tcg ttc gag tca cca tct tg-3′ (157 bp); Bmi-1 (forward)′-cca ggg ctt ttc aaa aat ga-3′ and (reverse) 5’-ccg atc caa tct gtt ctg gt-3′ (186 bp); Lgr5 (forward) 5′-ctc ttc ctc aaa ccg tct gc-3′ and (reverse) 5′-gat cgg agg cta agc aac tg-3′ (181 bp); CD44 (forward) 5′ – tgc cgc ttt gca ggt gta t-3′ and (reverse) 5′-ggc ctc cgt cc gaga ga-3′ (65 bp); CD133 (forward) 5′-gga ccc att ggc att ctc-3′ and (reverse) 5′-cag gac aca gca tag aat aat c-3′ (170 bp); Gli-1 (forward) 5′-cca tac atg tgt gag cac ga-3′ and (reverse) 5′-ggc aca gtc agt ctg ctt t-3′ (306 bp); Gli-2 (forward) 5′-caa cgc cta ctc tcc cag ac-3′ and (reverse) 5′-gag cct tga tgt act gta cca c-3′ (154 bp); SMO (forward) 5′-cat ccc tga ctg tga gat ca-3′ and (reverse) 5′-cac cat ctt ggt gac atg ct-3′ (369 bp); and GAPDH (forward) 5′-atc acc atc ttc cag gag cga-3′ and (reverse) 5′-agt tgt cat gga tga cct tgg c-3′ (283 bp). The qRT-PCR reaction and analysis were performed using CFX (Bio-Rad, Hercules, CA, USA).

### 4.6. Reporter Assay

For the reporter assay, HEK293T cells were plated in a 24-well plate, left to attach, and then transfected with 200 ng of reporters (TOPflash, HES, CSL, and Gli) together with 5 ng of renilla-luc (pRL-TK) plasmid using X-tremeGENE 9 DNA transfection reagent (Roche, Werk Penzberg, Germany). After 18 h of transfection, these cells were treated with 1-BP, 2-BP, 1,2-diBP, or DMSO (0.01%) and incubated 48 h at 37 °C and under 5% CO_2_. Luciferase activity was measured and normalized to renilla activity for transfection efficiency.

### 4.7. Statistical Analysis

Data are presented as the mean ± standard error of the mean (SEM) of 3 independent experiments with 3 replicates each. The statistical significance of the differences between groups was assessed using one-way analysis of variance followed by a post hoc Dunnett’s test for multiple comparisons. Statistical analysis was performed using GraphPad Prism software version 3.0 (GraphPad Software, Inc., La Jolla, CA, USA). *p* < 0.05 was considered to indicate a statistically significant difference.

## 5. Conclusions

In summary, low concentrations of BP compounds alter the expression of CSC markers in CRC cells and consequently increase their stemness. The ability of BP compounds to induce cancer stemness is regulated by cancer-related signaling pathways, including the Hedgehog and Wnt signaling pathways.
